# Buddhist culture as a safeguard for the subjective happiness of Chinese residents: mitigating anxiety regarding housing prices, unemployment, and inequality

**DOI:** 10.3389/fpsyg.2023.1282114

**Published:** 2023-12-14

**Authors:** Shuying Tan, Peijie Fang, Wenxiang Shi, Shukai Du

**Affiliations:** ^1^School of Economics and Finance, Chongqing University of Technology, Chongqing, China; ^2^School of Finance, Shanghai University of Finance and Economics, Shanghai, China

**Keywords:** happiness economics, subjective happiness, Buddhist culture, housing prices, unemployment, inequality

## Abstract

**Introduction:**

This study examines whether Buddhist culture in China can safeguard the subjective happiness of residents by mitigating the detrimental impact of adversity. Considering Chinese traditional culture and referencing Baidu Search Index data, we focus on three sources of anxiety that are currently troubling Chinese residents: housing prices, unemployment, and inequality.

**Methods:**

We conduct logit regressiontoinvestigate the mitigating impact of Buddhist culture on anxiety. The frequency of droughts and floods that occurred during the Ming and Qing dynasties are employed as instrumental variables for the local density of Buddhist culture to avoid endogeneity problems.

**Results:**

Empirical analysis based on microdata shows that Chinese Buddhist culture demonstrates the ability to alleviate the negative effects of housing price pressures, unemployment anxiety, and perceived inequality on subjective well-being. Mechanism analyses reveal that Chinese Buddhist culture plays a role in ameliorating the adverse impacts of housing and unemployment pressures on factors such as job satisfaction, physical health status, social trust, and expectations of future social standing. Moreover, it works to reduce inclinations toward social comparisons, thereby acting as a safeguard for happiness. Heterogeneity analysis shows that this insurance effect is more pronounced among vulnerable groups, including those in rural areas, middle-aged and elderly demographics, individuals with fewer social connections, lower social security coverage, and suboptimal health conditions.

**Discussion:**

This study expands the landscape of happiness economics research and provides novel evidence about the correlation between religion and happiness. Psychotherapists may draw on certain aspects of religious philosophy in addressing mental disorders. From a governmental perspective, there is potential to effectively steer religious culture towards fostering social harmony and promoting economic development.

## 1 Introduction

Happiness constitutes an unrelenting pursuit of humanity. While it might be commonly thought that an increase in income can amplify feelings of well-being, the *Easterlin Paradox* indicates that the ascent of individuals’ happiness levels may not necessarily mirror the rise in income (Easterlin et al., 2012). As the world’s second-largest economy, China’s *per capita* GDP surpassed $10,000 since 2019. However, the commensurate enhancement of well-being among Chinese residents has not mirrored the trajectory of income growth (Kahneman and Krueger, 2006). Embedded within Chinese traditional culture, “Living and working with contentment in a society of Great Harmony” encapsulates the Chinese people’s vision of a content and fulfilled life (Chen, 2014).^1^ This perspective on happiness is usually manifested through the pursuit of comfortable housing, dignified employment, and a fair and just environment. Hence, the pressures stemming from escalating housing prices, apprehensions of unemployment, and heightened perceptions of inequality can collectively widen the psychological distance to their envisioned ideal life, contributing to a decline in happiness.

**Figure 1:**
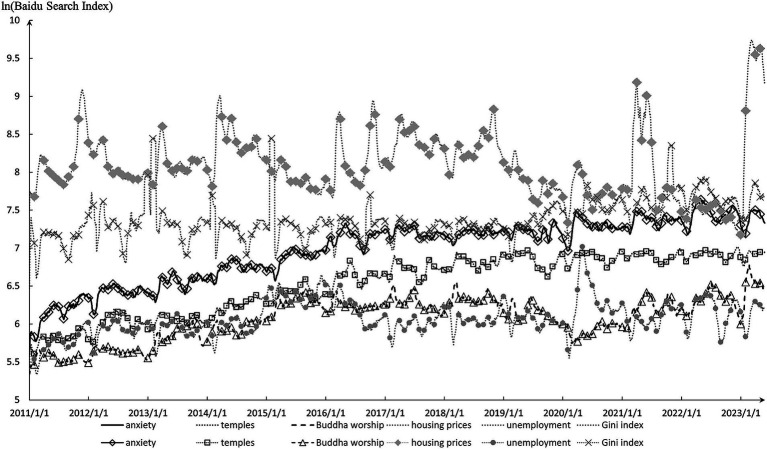
Baidu Search Index trend for keyword about anxiety and Buddhist culture. The magnitude of Baidu Search Index reflects the level of internet users’ attention towards searched keywords. The data is sourced from the official Baidu Index website.

As evidenced by Baidu Search Index data (Figure 1), the concerns of Chinese netizens regarding “anxiety” are rising and positively correlated with keywords like “housing prices,” “unemployment,” and “Gini coefficient” (Table 1). This implicates that the happiness of Chinese residents could be undermined by anxiety linked to housing prices, unemployment, and inequality. Buzzwords like *lying flat* and *Buddha-like*^2^ have gained substantial prominence across the Chinese internet.

**Table 1:** Correlation between Baidu Search Index of keywords.

	Anxiety	Temples	Buddha worship	Housing prices	Unemployment	Gini index
Anxiety	1					
Temples	0.943*	1				
Buddha worship	0.713*	0.688*	1			
Housing prices	0.101*	0.065*	0.311*	1		
Unemployment	0.449*	0.378*	0.291*	−0.063*	1	
Gini index	0.340*	0.269*	0.133*	0.014	0.290*	1

* denotes significance under 10% level.

Subjective happiness is correlated to both internal spirituality and external surroundings (Blanchflower, 2021; Kang and Kim, 2022). When external environment remains beyond an individual’s control, the key to retain happiness is to subjective emotions. Buddhism primarily aims to eliminate mental suffering. Building upon the principles and beliefs of Buddhism, some psychologists have suggested that it can play a role in alleviating sadness, reducing anxiety, and promoting overall subjective well-being (Fromm, 2013; Zhang, 2019). Therapies such as Mindfulness-Based Cognitive Therapy draw inspiration from Buddhist philosophy, assisting individuals in overcoming mental distress and achieving a state of happiness.

With a presence in China for over two millennia, Buddhism has ingrained itself as an integral facet of traditional Chinese culture, subtly shaping philosophical outlooks on life. Consequently, even though a vast majority of Chinese claim to have no religious beliefs, the Buddhist ideas can still cast an impact on their spiritual lives. The Baidu Search Index indicates a strong synchronicity among keywords “anxiety,” “temples,” and “Buddha worship” (Figure 1 and Table 1). Additionally, China’s news media frequently reports that the popularity of temple sites is increasing year by year. These social phenomena invite contemplation: amid the rapid thrust of economic development, do housing prices, unemployment, and inequality impede the well-being of Chinese residents? Furthermore, can Buddhist culture contribute to assuaging spiritual-level anxieties and sustaining individual subjective happiness?

This study is related to two strands of literature. First one focused on the factors that influence happiness, including housing price (Su et al., 2021; Liao et al., 2022; Shamsuddin and Campbell, 2022), unemployment (Winkelmann, 2009; Taht et al., 2020) and inequality (Schneider, 2016; Tavor et al., 2018). Most research findings indicate that a higher burden of housing costs, unemployment, and increased income inequality have a negative impact on happiness. Moreover, perceived inequality and the probability of job loss may be more detrimental to well-being (Schneider, 2016; Taht et al., 2020) than objective inequality or unemployment.

Another strand of literature explored the intricate connection between religion and happiness. Luttmer (2005) asserts that religion has positive impact on subjective well-being. Campante and Yanagizawa-Drott (2015) discover that while the religious practices have a negative influence on economic growth, they enhance the subjective well-being among religious adherents. Some scholars observed that religion can either intensify or alleviate a believer’s distress in response to diverse forms of tragic occurrences (Strawbridge et al., 1998; Hastings and Roeser, 2020).

These existing studies exhibit two primary limitations. First, the majority of attention has been directed towards religious countries, like Iran, Egypt (Campante and Yanagizawa-Drott, 2015), or Western nations, like the United States (Strawbridge et al., 1998; Luttmer, 2005; Hastings and Roeser, 2020). In these countries, there exists either an established official religion or a substantial proportion of individuals professing religious beliefs. In contrast, many Eastern countries are less developed compared to their Western counterparts. Furthermore, a distinctive characteristic of China is its classification as an “atheist” nation, with the majority of its populace identifying as irreligious.^3^ However, China is a country with a long history. Buddhism, as the predominant religion in the country,^4^ has been present in China for a considerable period, thereby weaving itself into the fabric of Chinese traditional culture. Consequently, Buddhism may wield a more imperceptible influence on the happiness of the Chinese people. Exploring the ways in which Buddhism influences happiness is a subject worthy of investigation.

Second, many studies have not addressed the issue of endogeneity in the regression of happiness on religion (Strawbridge et al., 1998; Luttmer, 2005; Hastings and Roeser, 2020). While religion might enhance happiness, it’s equally plausible that individuals turn to religion in times of adversity – implying that the influence of religion on happiness could be driven by reverse causation. Furthermore, the intricate interplay between housing prices, unemployment, and inequality could collectively shape the happiness of the Chinese population (Table 1). For example, rising housing prices might exacerbate inequality (Fuller et al., 2020). Thus, if the impact of inequality is not appropriately controlled, it could introduce omitted variable bias when analyzing the effect of housing prices on happiness.

Considering the limitations of the previous studies, this paper analyzes how housing price pressures, unemployment anxiety, and perceived inequality impact the happiness of Chinese residents, while also investigating the moderating role of Chinese Buddhist culture. Through empirical analysis using Chinese micro-level survey data, our findings demonstrate that housing price pressures, unemployment anxiety, and perceived inequality exert a negative influence on residents’ happiness. In regions characterized by a richer presence of Buddhist culture, the detrimental effects of housing and unemployment anxiety are effectively mitigated. This insurance effect on happiness is manifest across both Buddhist and non-Buddhist individuals in China. Mechanism analyses reveal that Chinese Buddhist culture plays a role in ameliorating the adverse impacts of housing and unemployment pressures on facets such as job satisfaction, physical health status, social trust, and future social standing expectations. Moreover, it operates to reduce inclinations towards social comparisons, thus acting as a safeguard for happiness. Heterogeneity analysis shows that this insurance effect is more salient among vulnerable groups, including those in rural areas, middle-aged and elderly demographics, individuals with fewer social connections, lower social security coverage, and suboptimal health conditions. This underscores the notion that Buddhist culture serves as a spiritual compensation for those confronting challenges in their real-world circumstances.

This paper contributes to the existing literature in three ways. First, it is the first one to investigate the relationship between Buddhism and happiness in China. The discovery of an insurance effect of religion, especially among non-Buddhists, suggests that the influence of Buddhism can extend to individuals who identify as “atheist.” Second, we innovatively harness the frequency of historical floods and droughts during the Ming and Qing dynasties as an instrumental variable for gauging local Buddhist culture density. Additionally, we control three happiness-detrimental factors in regression simultaneously to alleviate the potential endogeneity problem. Third, our study delves into the origins of the insurance effect of Buddhism. We propose that Buddhist culture in China fosters a serene mindset, effectively mitigating the adverse impacts of various pressures.

The remainder of this paper is organized as follows. Section 2 develops hypotheses. Section 3 presents data sources, descriptive analysis, and empirical methods. The baseline results, mechanism analysis, heterogeneous results and robustness checks are reported in Sections 4–6. Section 7 concludes the paper.

## 2 Theoretical analysis and hypothesis development

### 2.1 Housing prices, unemployment, inequality, and happiness

Housing holds a distinctive significance for the people of China. The swift escalation in housing prices within the nation has exacerbated the pressure on ordinary families to purchase houses or enhance their living conditions. Empirical research indicates that rising housing prices in China have a negative impact on happiness (Liao et al., 2022). To cope with soaring housing costs, the pursuit of a steady and sufficient income through gainful employment has emerged as a shared endeavor among numerous residents. Employment serves multifaceted purposes including social engagement, collective involvement, and identity affirmation (Jahoda, 1981), rendering it intrinsically tied to individuals’ overall sense of well-being. Individuals compelled to exit the labor market often experience a sense of deprivation and unhappiness (Di Tella et al., 2001). Even in the absence of actual unemployment, the surge in insecurity stemming from an elevated risk of unemployment can have an adverse impact on individuals’ happiness (Taht et al., 2020). This phenomenon is accentuated in contexts marked by high housing prices, as the loss of employment can compound the financial strains endured by typical households. Notably, China’s era of rapid housing price escalation has coincided with a period of robust economic growth. Paradoxically, this surge in economic prosperity did not yield a parallel increase in the happiness of Chinese citizens (Kahneman and Krueger, 2006). One plausible explanation for this disparity lies in the widening income inequality (Brockmann et al., 2009). Empirical research suggests that it is not necessarily the factual, but the perceived inequality that exerts an impact on individuals’ happiness (Alesina et al., 2004; Schwarze and Härpfer, 2007; Schneider, 2016). In other words, individuals’ happiness is influenced by inequality only if they perceive it as such.

Housing and employment significantly influence individuals’ subjective social status and quality of life. Fairness is a defining characteristic of an ideal society for many Chinese residents. The rapid escalation of housing prices increases the difficulty for ordinary families to achieve “comfortable living,” while growing unemployment anxiety hinders their ability to “enjoy work.” Additionally, the rise in perceived inequality amplifies a sense of deprivation among residents. Consequently, we develop the first hypothesis:

*H1:* The pressures of housing prices, unemployment anxiety, and perceived inequality have the potential to reduce the subjective happiness of Chinese residents.^5^

### 2.2 Core values of Buddhism and its impact on happiness

Religion holds the potential to shape individuals’ beliefs, attitudes, values, and even objective living conditions, thereby exerting both spiritual and material influences on happiness (Dehejia et al., 2007). However, the nature of this impact is multifaceted and not uniformly consistent (Clingingsmith et al., 2009; Campante and Yanagizawa-Drott, 2015). In general, most studies have demonstrated positive associations between religion and well-being (Koenig et al., 2012; Ramsay et al., 2019), and the mediators behind it have fallen into one of two categories: social resources and cognitive resources (Van Cappellen et al., 2016). Social resources such as participation in collective rituals and identification with religious groups are interpersonal in nature, suggesting that religious enhancement of well-being results from satisfaction of fundamental social needs for affiliation and relatedness (Smith, 1992; Ryan and Deci, 2000). Cognitive resources are more intrapersonal, pertaining to the way that religious belief influences an individual’s interpretation of their experiences. Religious or spiritual worldviews can imbue events with a sense of meaning or coherence by embedding them in larger cosmic narratives, which may in turn promote well-being. Religion can enhance positive emotions (Fredrickson, 2001, 2002). Religion can also function as a moderator of individuals’ emotional responses in the face of adversity, either mitigating the detrimental effects on their happiness or amplifying the distress they undergo (Pargament et al., 1990; Strawbridge et al., 1998; Hastings and Roeser, 2020). Hence, the influence of religion on happiness is contingent upon factors such as the country’s specific attributes, prevailing circumstances, and the inherent nature of the religion itself.

Buddhist culture stands as a significant facet of traditional Chinese culture, potentially exerting influence not solely over Buddhist adherents but also extending its impact to a wider spectrum of non-religious Chinese residents. The influence of Buddhist culture on Chinese residents might often go unnoticed by them, even as it remains objectively present. The principal objective of Buddhism lies in aiding individuals to transcend their afflictions and discover a life imbued with happiness and contentment. Central to Buddhism is the notion that the path to inner tranquility is paved through comprehending the concept of *Dependent Arising* and attaining a state of *Voidness*—wherein everything in life is ephemeral and in constant flux. This philosophy implicates that a life centered around material possessions does not yield lasting happiness, given their impermanent nature.^6^ Buddhism posits that human suffering is often rooted in greed, and in recognizing the transience of things, it advocates for a lighthearted approach towards material pursuits, desires, fame, and gain. This approach paves the way for a state of freedom, serenity, happiness, and liberation – both in times of favor and adversity. This facet of Buddhist ideology has also inspired psychological therapies such as *Mindfulness-Based Cognitive Therapy* and finds alignment with the tenets of *Morita Therapy*. Consequently, we proceed to formulate our second hypothesis:

*H2:* Buddhism aids individuals in comprehending the impermanent nature of all things and encourages a more balanced perspective towards the pursuits they may otherwise intensely struggle for. This capacity to embrace change and relinquish attachment can potentially mitigate the adverse effects of housing price pressures, unemployment anxiety, and perceived inequality on the subjective happiness of the Chinese residents.

## 3 Materials and methods

### 3.1 Data and sample

This study draws upon microdata sourced from the China Labor-force Dynamic Survey (CLDS), carried out by the Social Science Survey Center of Sun Yat-sen University, spanning the years 2014 and 2016. The survey specifically targets individuals aged 15–64 and encompasses an array of themes, ranging from respondents’ psychological well-being and employment status to their health condition and family circumstances. The sample encompasses 29 provinces across China, thereby ensuring national representativeness. In contrast to frequently employed Chinese microdatasets like the China Household Finance Survey (CHFS) or the China Family Panel Studies (CFPS), CLDS offers researchers an added advantage of accessing intricate details at city level.^7^ After excluding invalid observations, a total of 19,072 valid samples were garnered for analysis.

### 3.2 Measures

#### 3.2.1 Dependent variable: subjective happiness

The CLDS questionnaire includes a question that asks respondents to rate their overall life happiness on a scale from 1 to 5, with options representing “extremely unhappy,” “unhappy,” “neutral,” “happy,” and “extremely happy,” respectively. In this study, we transformed the responses into a binary variable indicating happiness or unhappiness. Specifically, if respondents selected “happy” or “extremely happy,” the variable was assigned a value of 1; otherwise, it received a value of 0. This transformation was employed due to respondents’ tendency to avoid extreme answers when using five-point Likert scales. Including the original five-point scale directly in the analysis may lead to central tendency bias. Converting the responses into a 0–1 variable helps alleviate this issue to some extent and is more suitable for studying the state of happiness rather than its intensity (Bertrand, 2013).^8^

#### 3.2.2 Independent variable: housing price pressure, unemployment anxiety and perceived inequality

The housing price pressure within a city is computed as the growth rate of the ratio of housing prices to *per capita* disposable income. Our assertion is that rapid surges in housing prices, rather than the absolute level of housing prices, are more likely to evoke anxiety among residents. Even if housing prices are relatively elevated, a gradual increment might afford residents the expectation that diligent effort and prudent saving could eventually alleviate the anxiety stemming from high housing costs. However, in instances of swift and dramatic housing price escalation, residents could perceive that their endeavors are struggling to keep pace with the rapid price hikes, resulting in heightened anxiety.

Building on existing research (Taht et al., 2020), this study primarily employs respondents’ subjective perceptions to measure their anxiety related to unemployment and perceived inequality. Respondents were asked to indicate their level of agreement with the statement: “How likely is it that you will experience unemployment in the future?” The responses were transformed into a binary variable: a response of “very likely” or “somewhat likely” was coded as 1, while other responses were coded as 0. Perceived inequality was assessed based on respondents’ agreement with the statement: “Do you think your current living standard is fair in comparison to your efforts at work?” Responses indicating “completely unfair” or “somewhat unfair” were coded as 1, while other responses were coded as 0.

#### 3.2.3 Independent variable: density of Buddhist culture

In this study, we measure the density of Buddhist culture by the number of Buddhist temples per square kilometer in the city in 2014.^9^ It is important to acknowledge that precisely quantifying the number of Buddhist followers in China presents challenges, particularly when compared to other religions like Islam, Catholicism, etc., which tend to have more well-defined formal entry procedures. Consequently, there might be individuals who are deeply influenced by Buddhist culture but do not identify themselves as Buddhist followers. Thus, there might be individuals who are significantly influenced by Buddhist culture yet do not identify themselves as official Buddhist adherents. Therefore, utilizing respondents’ direct responses to a straightforward survey question such as “Do you believe in religion?” might not accurately capture the extent of Buddhist culture’s impact on them.

However, individuals’ beliefs and behavior are often shaped by their surroundings. The prevalence of Buddhist temples is indicative of investments from either followers or official sources, both of which are linked to the density of Buddhist culture in the area. The existence of Buddhist temples also functions as a means of propagating Buddhist culture. Consequently, a greater concentration of Buddhist temples can reasonably serve as an indicator of a more pronounced density of Buddhist culture. This suggests the likelihood of a more substantial impact on the well-being of local residents in areas with higher temple density.

#### 3.2.4 Other controls

Grounded in prior research, this study controls for individual, household, and city-level characteristics to mitigate omitted variable bias (Luttmer, 2005; Dynan and Ravina, 2007; Campante and Yanagizawa-Drott, 2015; Ding et al., 2021; Qiao and Cai, 2023). The individual-level characteristic variables include: *Gender*, with males coded as 1 and females as 0; *Age*, where respondents outside the age range of 15–65 years are excluded based on survey guidelines; *Marital status*, with married individuals coded as 1 and others as 0; *Ethnicity*, with Han ethnicity coded as 1 and ethnic minorities as 0; *Self-rated health status*, respondents who consider themselves as “healthy” or “very healthy” are coded as 1, while others are coded as 0.; *Political affiliation*, with party members coded as 1 and non-party members as 0; *Weekly working hours*; *Education*, with values ranging from 1 to 9 representing no schooling, primary or private school, junior high school, regular high school, vocational high school, technical school, secondary specialized school, junior college, and undergraduate or higher, respectively.

Household-level characteristic variables include: *Household size*, measured by the number of permanent residents in the household; *Homeownership*, coded as 1 for households with self-owned property and 0 otherwise; *Annual absolute household income*; *Annual relative household income*, calculated as the difference between the household’s absolute income and the average absolute income of households within the same county; *Share of food expenditure in the household budget*, equal to the ratio of food consumption to total consumption; *Number of children*, representing the count of family members aged below 14 years.

City-level characteristic variables include *GDP growth rate*, CPI, *Proportion of tertiary industry output*, and *Number of permanent residents in the city*. Descriptive statistics for these variables are presented in Table 2.

**Table 2:** Descriptive statistics.

Variable	Unit	Count	Mean	SD	Min	Max
**Dependent**						
Subjective happiness	1	19,072	0.61	0.49	0	1
**Independent**						
Housing price pressure	1	19,072	0.01	0.12	−0.22	0.63
Unemployment anxiety	1	19,072	0.14	0.35	0	1
Perceived inequality	1	19,072	0.23	0.42	0	1
Density of Buddhist culture	1/km^2^	19,072	0.55	1.23	0.002	7.2
Frequency of floods and droughts	100	18,481	2.21	0.66	0.86	3.3
**Controls**						
**Individual-level**						
Gender	1	19,072	0.53	0.50	0	1
Age	1	19,072	43.96	11.74	15	65
Marriage	1	19,072	0.85	0.36	0	1
Ethnicity	1	19,072	0.93	0.26	0	1
Party member	1	19,072	0.08	0.28	0	1
Health	1	19,072	0.62	0.48	0	1
Working hours	hour	19,072	45.03	21.67	0	98
Education	1	19,072	3.68	2.33	1	9
**Household-level**						
Household size	1	19,072	4.56	1.92	1	20
Homeownership	1	19,072	0.84	0.37	0	1
Absolute income	10,000¥	19,072	5.88	5.35	0.001	40
Relative income	10,000¥	19,072	0.28	4.71	−13	36
Food expenditure	1	19,072	0.40	0.24	0	1
Number of children	1	19,072	0.67	0.86	0	8
**City-level**						
GDP growth	1	19,072	0.07	0.06	−0.35	0.16
CPI	1	19,072	102	0.51	101	103
Tertiary output proportion	1	19,072	0.45	0.11	0.23	0.82
Population	10^6^	19,072	5.83	4.11	1.1	34

### 3.3 Empirical strategy

This study employs the Logit regression, as shown in Eq. 1, to examine the effects of housing price pressure, unemployment anxiety, and perceived inequality on individuals’ happiness, as well as the moderating role of Buddhist culture.


(1) $$\begin{array}{l} \mathrm{Pr}\left(happiness_{i}=1\right)=\alpha +\beta_{1}hprs_{c}+\beta_{2}uemp_{i}+\beta_{3}ueql_{i}\\ \;\;\;\;\;\;\;\;\;\;\;\;\;\;\;\;\;\;\;\;\;\;\;\;+\gamma_{0}rel_{c}+\gamma_{1}hprs_{c}\times rel_{c}+\gamma_{2}uemp_{i}\times rel_{c}\\ \;\;\;\;\;\;\;\;\;\;\;\;\;\;\;\;\;\;\;\;\;\;\;\;+\gamma_{3}ueql_{i}\times rel_{c}+\tau^{\prime }x+\varepsilon_{i} \end{array}$$

where $happiness_{i}$ is a dummy indicating happiness status (0 or 1) of respondent i , $hprs_{c}$ denotes housing price pressure in the city where respondent i resides, $uemp_{i}$ captures respondent $i^{’}$ s level of unemployment anxiety, $ueql_{i}$ measures respondent $i^{’}$ s perception of inequality, and $rel_{c}$ stands for the density of Buddhist culture in the city where respondent i lives. The vector x encompasses individual characteristics, household conditions, city development level, and year fixed effects. Based on theoretical analyses, we expect that ${\overset{}{\hat{\beta }}}_{i}<0,{\overset{}{\hat{\gamma }}}_{j}>0$ , for i,j∈{1,2,3} .

### 3.4 Endogeneity issue and instrument variable

Potential endogeneity issues may exist if individuals seeking higher levels of happiness actively choose to reside in areas with a higher density of Buddhist culture. Moreover, cities with a higher density of Buddhist culture may attract more talents, financial resources, which can contribute to residents’ feelings of happiness. To address these concerns, this study employs the frequency of drought and flood disasters that occurred in each region during the Ming and Qing dynasties as an instrumental variable for the local density of Buddhist culture.^10^

On one hand, the frequency of drought and flood disasters during the Ming and Qing dynasties shows a weak relationship with city attractiveness after 2014, satisfying the exclusivity requirement of instrumental variables. On the other hand, in eras or regions where natural sciences were less developed, people often interpreted disasters such as floods, droughts, and earthquakes as divine retribution. Religious culture serves as consolation for individuals facing overwhelming adversities, and the number and prominence of religious sites reflected the fervor of people’s faith. Evidence from various countries demonstrates that after disasters occurred, people’s devotion to religion increased, leading to the expansion of religious rituals and the growth in both the number and scale of religious establishments (Belloc et al., 2016). This study posits that in regions where droughts and floods were more frequent during the Ming and Qing dynasties, people were more likely to turn to traditional Chinese religious culture, including Buddhism, for comfort and blessings. Consequently, a higher count of Buddhist temples would exist, meeting the relevance condition for instrumental variables.

## 4 Empirical results

### 4.1 Baseline results

Table 3 reports the average marginal effects obtained from model (1) estimated by Logit regression. As shown in columns (1)–(3), after controlling for a series of covariates, the coefficients of housing price pressure, unemployment anxiety, and perceived inequality are all significantly negative. This points to the fact that concerns revolving around housing prices, unemployment, and inequality contribute to a decrease in the subjective happiness of Chinese residents. This supports our hypothesis *H1*.

**Table 3:** The insurance effect of Buddhist culture in safeguarding happiness.

Dependent variable	Subjective happiness			
Method	Logit			IV-probit
	(1)	(2)	(3)	(4)
Housing price pressure	−0.141***	−0.120***	−0.199***	−0.178***	(0.000)	(0.000)	(0.000)	(0.000)
Unemployment anxiety	−0.100***	−0.068***	−0.080***	−0.097***	(0.000)	(0.000)	(0.000)	(0.000)
Perceived inequality	−0.215***	−0.183***	−0.183***	−0.180***	(0.000)	(0.000)	(0.000)	(0.000)
Buddhist culture			0.013***	0.011**			(0.000)	(0.041)
HP pressure × Buddhist culture			0.135***	0.085**
			(0.000)	(0.021)
Unemployment × Buddhist culture			0.021***	0.045***
			(0.007)	(0.000)
Inequality × Buddhist culture			−0.001	−0.007
			(0.926)	(0.515)
Gender		−0.020***	−0.021***	−0.019***		(0.004)	(0.003)	(0.007)
Age		−0.009***	−0.009***	−0.009***		(0.000)	(0.000)	(0.000)
Age square		0.000***	0.000***	0.000***		(0.000)	(0.000)	(0.000)
Married		0.064***	0.063***	0.062***		(0.000)	(0.000)	(0.000)
Ethnicity		0.022*	0.017	0.021		(0.095)	(0.206)	(0.120)
Party member		0.091***	0.089***	0.087***		(0.000)	(0.000)	(0.000)
Health		0.154***	0.153***	0.154***		(0.000)	(0.000)	(0.000)
Working hours		0.000	0.000	0.000		(0.500)	(0.357)	(0.323)
Education		0.005***	0.006***	0.005***		(0.009)	(0.004)	(0.005)
Family size		−0.011***	−0.011***	−0.011***		(0.000)	(0.000)	(0.000)
Own house		0.062***	0.053***	0.055***		(0.000)	(0.000)	(0.000)
Absolute income		0.075	0.058	0.061		(0.144)	(0.256)	(0.231)
Absolute income square		−0.004	−0.003	−0.003		(0.193)	(0.350)	(0.319)
Relative income		0.000***	0.000***	0.000***		(0.000)	(0.000)	(0.000)
Food expenditure		−0.073***	−0.068***	−0.070***		(0.000)	(0.000)	(0.000)
Number of children		0.002	0.001	0.001		(0.667)	(0.852)	(0.814)
GDP growth		−0.130**	−0.171***	−0.193***		(0.027)	(0.004)	(0.002)
CPI		0.012	0.023***	0.020***		(0.112)	(0.002)	(0.010)
Tertiary output proportion		0.003***	0.003***	0.003***		(0.000)	(0.000)	(0.000)
City population		−0.012**	−0.012**	−0.013**		(0.041)	(0.039)	(0.028)
Observations	19,072	19,072	19,072	18,569
Year FE	Yes	Yes	Yes	Yes
First stage estimation of IV regression				
Variable		Buddhist culture		
Frequency of floods and droughts		0.855***		
		(0.000)		

Coefficients represent average marginal effects. Values in parentheses are *p*-values; *p-value < 0.1, **p-value < 0.05m, ***p-value < 0.01. The Anderson canon. Corr. LM statistic for under identification test is 436.333, and the Cragg-Donald Wald F statistic for weak identification test is 89.351.

The coefficient linked to Buddhist culture density and its interaction with housing price pressure and unemployment anxiety are notably positive. This suggests that regions characterized by a higher density of Buddhist culture encounter a relatively lesser negative impact of housing price pressure and unemployment anxiety on the subjective happiness of individuals. This could be attributed to the core values of Buddhism that help people take lightly their concerns and navigate through hardships serenely. However, the interaction between Buddhist culture density and perceived inequality is not significant, indicating that Buddhist culture does not mitigate the negative impact of perceived inequality on subjective happiness among Chinese residents. This is possibly due to the emphasis on “Existence Being Equal” within Buddhism. Overall, these results suggest that Chinese Buddhist culture has an insurance effect in safeguarding happiness, which supports our hypothesis H2.

Column (4) presents the second-stage results of the IV-probit estimation using the frequency of drought and flood disasters during the Ming and Qing dynasties as the instrumental variable for Buddhist culture density. The signs and significance of variables of interest remain consistent. The first-stage outcomes reveal that cities experiencing more drought and flood disasters during the Ming and Qing dynasties manifest elevated levels of Buddhist culture density, aligning with theoretical expectations and allowing for the rejection of the weak instrumental variable hypothesis. The resilience of the instrumental variable regression outcomes provides reassurance against concerns of endogeneity associated with our measurement of Buddhist culture density.

The estimated coefficients of control variables indicate that females report higher levels of happiness on average. The coefficient of age is significantly negative, while the coefficient of age squared is significantly positive, indicating that middle-aged individuals have the lowest average level of happiness, which aligns with the concept of “midlife crisis.” Married individuals, on average, report higher levels of happiness, suggesting that marriage contributes positively to well-being. Higher educational attainment is associated with increased happiness. On average, having more family members reduces happiness. Owning a house implies greater stability in life, which is beneficial for increased happiness. Relative income increase has a more positive impact on happiness compared to absolute income. Higher food expenditure as a proportion of household consumption is associated with lower happiness, indicating that households with higher Engel coefficients experience lower subjective happiness. Furthermore, residents in regions with a greater proportion of tertiary industry output report higher levels of happiness. Conversely, individuals residing in areas with larger populations tend to exhibit lower levels of happiness. Interestingly, the negative coefficient of the GDP growth rate implies that rapid economic growth does not necessarily translate into higher happiness levels. This indirectly aligns with findings by Easterlin et al. (2012).

### 4.2 Mechanism behind the insurance effect of Buddhist culture

The baseline findings indicate that in response to the pressures of housing prices and unemployment anxiety, Buddhist culture acts as a protective factor for individuals’ subjective happiness. To gain a deeper understanding of how this protective effect operates, we employ a two-step methodology. In the first step, we explore the mechanisms through which housing price pressure, unemployment anxiety, and perceived inequality impact individuals’ subjective well-being. Subsequently, in the second step, we investigate whether Buddhist culture mitigates or amplifies these mechanisms, as outlined in model (2, 3).

(2) $$\begin{array}{l} channel_{i}=a+b_{1}hprs_{c}+b_{2}uemp_{i}+b_{3}ueql_{i}+c_{0}rel_{c}\\ \;\;\;\;\;\;\;\;\;\;\;\;\;\;\;\;\;\;\;\;\;\;+c_{1}hprs_{c}\times rel_{c}+c_{2}uemp_{i}\times rel_{c}\\ \;\;\;\;\;\;\;\;\;\;\;\;\;\;\;\;\;\;\;\;\;\;+c_{3}ueql_{i}\times rel_{c}+\phi^{\prime }x+\varepsilon_{i} \end{array}$$

(3) $$\begin{array}{l} \mathrm{Pr}\left(happiness_{i}=1\right)=\alpha +\beta_{0}channel_{i}+\beta_{1}hprs_{c}+\beta_{2}uemp_{i}\\ \;\;\;\;\;\;\;\;\;\;\;\;\;\;\;\;\;\;\;\;\;\;\;\;\;\;\;\;\;\;\;\;\;\;\;\;\;\;\;\;\;\;\;\;\;+\beta_{3}ueql_{i}+\gamma_{0}rel_{c}+\gamma_{1}hprs_{c}\times rel_{c}\\ \;\;\;\;\;\;\;\;\;\;\;\;\;\;\;\;\;\;\;\;\;\;\;\;\;\;\;\;\;\;\;\;\;\;\;\;\;\;\;\;\;\;\;\;\;+\gamma_{2}uemp_{i}\times rel_{c}+\gamma_{3}ueql_{i}\times rel_{c}+\tau^{\prime }x+\upsilon_{i} \end{array}$$

If a certain factor is positively associated with happiness ( $\beta_{0}$ is positive), but housing price, unemployment, or inequality anxieties exert negative impacts on the it ( $b_{1},b_{2},b_{3}$ are negative), it suggests that these three anxiety factors may reduce happiness through this specific mechanism. In this scenario, if Buddhist culture assists in mitigating the adverse effects of anxiety factors on the variables associated with this mechanism ( $c_{1},c_{2},c_{3}$ are positive), it implies that Buddhist culture acts as a safeguard for happiness by impeding the mechanism through which these negative impacts are propagated.

#### 4.2.1 Job satisfaction

Work serves not only as a means of livelihood but also as a pathway to social recognition and personal development (Jahoda, 1981). The level of job satisfaction plays a crucial role in determining individuals’ overall happiness (Piccolo et al., 2005; Booth and Van Ours, 2008). Religious culture helps residents perceive divine meaning, purpose, and significance in relatively mundane daily events, and such interpretations associate positively with positive emotions and appraisals of well-being (Ramsay et al., 2019). In the face of high housing prices, the preferences of workers may become distorted, prioritizing salary levels over personal preferences. This can result in limited job choices and reluctance to explore alternative employment options, even if they are dissatisfied with their current job. In this study, respondents’ answers to the question “How satisfied are you with your current/last job overall?” are employed as an indicator of their job satisfaction. Responses indicating “very satisfied” or “somewhat satisfied” are assigned a value of 1, while other responses are assigned a value of 0.

The regression results presented in Table 4, column (1), reveal that an increase in job satisfaction contributes to higher levels of happiness, whereas the escalation of housing price pressure significantly diminishes job satisfaction. Nevertheless, Buddhist culture acts as a mitigating factor, tempering the adverse influence of housing price pressure on job satisfaction and alleviating the decline in happiness associated with it. This ameliorating effect can be attributed to the philosophical tenets of “letting go” within Buddhist culture, which aids individuals in loosening their attachment to material possessions, as well as the principle of “letting nature take its course,” empowering people to confront adversity and dissatisfaction with a serene mindset while continuing to exert their best efforts.

**Table 4:** The mechanism behind the insurance effect of Buddhist culture.

	(1)	(2)	(3)	(4)	(5)
	Job satisfaction	Expected social strata	Health	Sense of trust	Comparison psychology
Panel A: Channels that anxiety factors influence happiness					
Housing price pressure	−0.186***	−0.100	−0.138	−0.078***	−0.190***	(0.000)	(0.445)	(0.717)	(0.002)	(0.000)
Unemployment anxiety	−0.012	−0.367***	−0.522***	−0.061***	−0.157***	(0.303)	(0.000)	(0.000)	(0.000)	(0.000)
Perceived inequality	−0.153***	−0.690***	−0.571***	−0.094***	−0.262***	(0.000)	(0.000)	(0.000)	(0.000)	(0.000)
Buddhist culture	0.031***	−0.003	−0.079	−0.004	−0.001	(0.000)	(0.855)	(0.118)	(0.227)	(0.874)
HP pressure × Buddhist culture	0.128***	0.198*	0.580***	0.044**	0.095**	(0.000)	(0.054)	(0.001)	(0.033)	(0.012)
Unemployment × Buddhist culture	−0.002	0.055*	0.080***	0.000	0.023*	(0.772)	(0.078)	(0.000)	(0.959)	(0.071)
Inequality × Buddhist culture	0.000	0.044*	0.049*	0.000	0.014	(0.970)	(0.080)	(0.054)	(0.938)	(0.140)
Panel B: Buddhist culture moderate the channels					
Job satisfaction	0.211***					(0.000)				
Expected social strata		0.044***					(0.000)			
Health			0.153***					(0.000)		
Sense of trust				0.085***					(0.000)	
Comparison psychology					0.100***					(0.000)
Housing price pressure	−0.160***	−0.194***	−0.199***	−0.191***	−0.184***	(0.000)	(0.000)	(0.000)	(0.000)	(0.000)
Unemployment anxiety	−0.079***	−0.064***	−0.080***	−0.074***	−0.069***	(0.000)	(0.000)	(0.000)	(0.000)	(0.000)
Perceived inequality	−0.149***	−0.153***	−0.183***	−0.174***	−0.171***	(0.000)	(0.000)	(0.000)	(0.000)	(0.000)
Buddhist culture	0.005	0.013***	0.013***	0.013***	0.013***	(0.130)	(0.000)	(0.000)	(0.000)	(0.000)
HP pressure×Buddhist culture	0.107***	0.126***	0.135***	0.130***	0.124***	(0.000)	(0.000)	(0.000)	(0.000)	(0.000)
Unemployment × Buddhist culture	0.020***	0.018**	0.021***	0.021**	0.019**	(0.008)	(0.019)	(0.008)	(0.011)	(0.020)
Inequality × Buddhist culture	−0.000	−0.002	−0.001	−0.001	−0.002	(0.994)	(0.710)	(0.929)	(0.922)	(0.758)
Other controls	Yes				
Year FE	Yes				
Observations	18,682	19,072	19,072	19,072	19,072

Coefficients represent average marginal effects. Values in parentheses are *p*-values; *p-value < 0.1, **p-value < 0.05, ***p-value < 0.01.

Furthermore, the findings from column (1) of Table 4 also indicate that an increase in perceived income inequality results in a decrease in job satisfaction. However, Buddhist culture does not alleviate the negative impact of perceived income inequality on job satisfaction. This outcome may arise from the fact that although Buddhist principles inherently embrace the concept of equality among all sentient beings, they may not yield a substantial mitigating effect on the decline in happiness induced by perceived inequality.

#### 4.2.2 Expected social strata in the future

Lots of people have anxiety related to social status (Botton, 2005; Haller and Hadler, 2006). Anticipations of future social strata can impact an individual’s sense of happiness (Gallo and Matthews, 2003). Those with improved housing conditions are more likely to align themselves with a higher social echelon. However, the swift escalation of housing prices can hinder the attainment of favorable housing, potentially dampening the prospects of ascending the social ladder for the general populace. Traditional Chinese beliefs also correlate stable and esteemed employment with higher social standing. Thus, heightened concerns of job loss among residents could indicate a greater likelihood of envisioning a decline in future social status. The perception of inequality similarly influences perceptions of social mobility. Individuals might attribute inequality to external factors such as societal structures, culminating in pessimistic anticipations about their prospects for upward mobility. In this study, respondents’ answers to the question “Where do you expect to be positioned in society five years from now?” (with “10” symbolizing the highest level and “1” signifying the lowest) were employed to gauge their outlook on future social strata.

The outcomes displayed in column (2) of Table 4 reveal that both unemployment anxiety and perceived inequality are linked to adverse anticipations regarding future social positions among Chinese residents, consequently exerting a detrimental impact on their happiness. Nevertheless, Buddhist culture plays a role in alleviating these negative expectations, showcasing its capacity to safeguard happiness.

#### 4.2.3 Health

Sub-health conditions contribute to a negative impact on subjective well-being (Cattaneo et al., 2009). Prior research shows that religious people tend to have a relatively healthy lifestyle (Eid and Larsen, 2008). The escalation of housing prices often leads to heightened stress levels. The experience of unemployment tends to generate feelings of deprivation (Voßemer et al., 2018). Consequently, both housing price pressure and unemployment anxiety may yield a detrimental impact on an individual’s health. Additionally, individuals’ negative emotional reactions, such as anger and resentment, can arise when they perceive a disparity between their possessions and their perceived entitlements. Consequently, the perceived inequality may be detrimental to health. Within this study, respondents’ self-rated health status was utilized as a binary variable (coded as 0 or 1) to assess their physical health condition.

The results presented in column (3) of Table 4 demonstrate that increased unemployment anxiety and perceived inequality are detrimental to an individual’s health, while Buddhist culture helps alleviate the adverse effects, thus providing a safeguarding effect on subjective well-being. This suggests that Buddhist culture can assist in maintaining health and enhance an individual’s subjective well-being.

#### 4.2.4 Sense of trust

Social trust has been identified as a significant determinant of individuals’ subjective well-being (Lu et al., 2020). The influences on social trust can be categorized into two main factors: personal characteristics and the external environment (Warren, 1999). Personal attributes encompass aspects like the sense of control, optimism, and religious beliefs, while the external environment includes factors such as the quality of public services and income inequality. The pressures of increased housing prices and unemployment anxiety can erode individuals’ sense of control over their lives, leading to a reduction in their overall level of social trust. Additionally, income inequality can spur individuals to make comparisons about their own living standards, which can engender feelings of exclusion and jealousy, ultimately fostering an atmosphere of mistrust among individuals and negatively impacting their overall well-being.

The results presented in column (4) of Table 4 demonstrate that anxieties stemming from housing price, unemployment, and inequality contribute to a rise in individuals’ sense of distrust. However, the presence of Buddhist culture acts as a mitigating factor, reducing the negative impact of housing price pressure on trust. This phenomenon could be attributed to the compassionate and altruistic principles inherent in Buddhist teachings. When individuals face various pressures, especially those related to housing prices, and their perception of control over their lives diminishes, Buddhist culture can serve as a regulator for their emotional state. It guides them towards adopting a more compassionate and trusting outlook towards the people and circumstances around them. In doing so, Buddhist culture acts as a safeguard for their subjective well-being.

#### 4.2.5 Social comparison psychology

Individuals strive to maintain parity with their peers in terms of lifestyle, possessions, or status. In this study, we label the resulting decline in subjective well-being due to the perception of one’s life as being inferior to that of others as social comparison psychology (Esping-Andersen and Nedoluzhko, 2017; Huang and Fang, 2021). For everyday residents, the increase in housing price pressure adds complexity to the pursuit of high-quality housing, heightened unemployment anxiety complicates efforts to sustain a decent standard of living, and growing income inequality intensifies sensations of deprivation. As a result, the anxieties related to housing prices, unemployment, and inequality are likely to diminish happiness by fueling social comparison psychology. To gauge respondents’ engagement in social comparison psychology, the questionnaire included a query: “In comparison to your neighbors, do you believe your current living standard is higher or lower?” Responses indicating “higher” or “much higher” were scored as 1, while other responses were assigned a score of 0.

The results presented in column (5) of Table 4 demonstrate that the coefficients of housing price, unemployment, and inequality anxieties are all significantly negative, indicating that these anxieties lead residents to perceive their lives as inferior to those around them, resulting in reduced happiness. The coefficients of the interaction terms between Buddhist culture and housing price, and between Buddhist culture and unemployment, are both positive, suggesting that Buddhist culture helps alleviate social comparison psychology and thus mitigates the negative impact of anxiety factors on happiness. This phenomenon could be attributed to the inherent principles within Buddhism that encourage individuals to consider the happiness of others as tantamount to their own, thereby diminishing the inclination towards comparing oneself unfavorably to others and fostering a sense of serenity and contentment.

The findings from the outlined mechanism analysis reveal that housing price pressure, unemployment anxiety, and perceived inequality have distinct and negative impacts on different aspects, including job satisfaction, expectations about future social status, physical health, societal trust, and the sense of deprivation arising from social comparison. These combined effects contribute to a reduction in overall subjective well-being. Notably, in accordance with the Buddhist principle of “Existence Being Equal,” Buddhist culture exerts a more pronounced inhibitory influence on the mechanisms that connect housing price and unemployment anxieties to the decline in happiness. In contrast, its mitigating effect on the mechanism linking inequality anxiety to happiness is comparatively more modest.

## 5 Heterogeneity analysis

The impact of housing price pressure, unemployment anxiety, and inequality perception on individual happiness may vary due to differences in individual characteristics and environmental factors. Consequently, the protective effect of Buddhist culture on happiness may also exhibit heterogeneity.

### 5.1 Urban and rural area

In certain rural areas of China, a phenomenon known as “religious culture fervor” is present. In the context of this study, we partitioned the sample into urban and rural segments based on the residential locations of the respondents. The findings are displayed in Table 5, columns (1) and (2). For rural inhabitants, Buddhist culture exhibits not only a more significant direct impact on happiness but also a more prominent alleviating effect on housing price pressure and unemployment anxiety.

**Table 5:** Heterogeneous effect of Buddhist culture.

	(1)	(2)	(3)	(4)	(5)	(6)	(7)	(8)	(9)	(10)	(11)
	Urban	Rural	Young	Middle-age	Elderly	Many friends	Few friends	With medical insurance	Without medical insurance	Good Health	Sub-health
Housing price pressure	−0.183***	−0.217***	−0.250***	−0.163***	−0.194***	−0.200***	−0.182***	−0.087	−0.242***	−0.174***	−0.262***	(0.001)	(0.000)	(0.000)	(0.001)	(0.003)	(0.000)	(0.001)	(0.106)	(0.000)	(0.000)	(0.000)
Unemployment anxiety	−0.117***	−0.047***	−0.103***	−0.088***	−0.045**	−0.078***	−0.063***	−0.117***	−0.056***	−0.081***	−0.078***	(0.000)	(0.001)	(0.000)	(0.000)	(0.026)	(0.000)	(0.000)	(0.000)	(0.000)	(0.000)	(0.000)
Perceived inequality	−0.165***	−0.192***	−0.157***	−0.183***	−0.217***	−0.198***	−0.175***	−0.172***	−0.190***	−0.168***	−0.210***	(0.000)	(0.000)	(0.000)	(0.000)	(0.000)	(0.000)	(0.000)	(0.000)	(0.000)	(0.000)	(0.000)
Buddhist culture	−0.012*	0.017***	0.008	0.011**	0.019***	0.009	0.016***	0.003	0.014***	0.006	0.021***	(0.078)	(0.000)	(0.277)	(0.037)	(0.002)	(0.136)	(0.006)	(0.783)	(0.000)	(0.221)	(0.000)
HP pressure × Buddhist culture	0.017	0.164***	0.157***	0.106***	0.164***	0.100**	0.122***	0.086	0.150***	0.122***	0.171***
	(0.740)	(0.000)	(0.001)	(0.007)	(0.002)	(0.020)	(0.005)	(0.126)	(0.000)	(0.000)	(0.000)
Unemployment × Buddhist culture	0.006	0.020**	0.028*	0.026**	0.003	0.009	0.024**	0.011	0.018**	0.013	0.026**
	(0.652)	(0.035)	(0.099)	(0.017)	(0.840)	(0.480)	(0.049)	(0.486)	(0.042)	(0.230)	(0.020)
Inequality × Buddhist culture	0.015	−0.004	−0.005	0.003	−0.000	0.012	−0.002	0.006	−0.002	0.005	−0.003	(0.186)	(0.579)	(0.665)	(0.709)	(0.976)	(0.248)	(0.798)	(0.621)	(0.820)	(0.584)	(0.702)
Observations	10,186	8,886	5,679	7,972	5,421	7,117	6,939	6,269	12,803	11,914	7,158
Other controls	Yes	Yes	Yes	Yes	Yes	Yes	Yes	Yes	Yes	Yes	Yes
Year FE	Yes	Yes	Yes	Yes	Yes	Yes	Yes	Yes	Yes	Yes	Yes

Coefficients represent average marginal effects. Values in parentheses are *p*-values; *p-value < 0.1, **p-value < 0.05, ***p-value < 0.01.

This finding provides additional evidence of the “religious culture fervor” phenomenon in rural China, which may be attributed to three factors. First, religious cultural activities have a crowding-out effect on economic production (Campante and Yanagizawa-Drott, 2015), and urban areas typically have higher productivity compared to rural areas. Thus, rural residents may face lower opportunity costs in engaging in religious cultural activities. Second, rural China is characterized by a “Guanxi” society where social relationships play a crucial role. Religious cultural activities not only provide spiritual comfort to rural residents but also serve as a medium for interpersonal interactions, facilitating the accumulation of social capital. Third, rural areas often lack adequate social security and public cultural resources, making religious cultural activities a means for rural residents to share risks and enrich their spiritual lives.

### 5.2 Age

Individuals in different age groups face varying types and levels of life pressures, leading to diverse pursuits and experiences in life. Based on the age distribution of the questionnaire sample, we categorized individuals under the age of 35 as “young,” those between 35 and 50 years old as “middle-aged,” and those aged 50 and above as “elderly.”

The findings presented in Table 5, columns (5) and (6), highlight distinct patterns in the factors affecting happiness among different age groups. Specifically, decreased happiness among young individuals appears to be primarily linked to housing price pressure and unemployment anxiety, while for the elderly, a significant contributor is a sense of inequality. Meanwhile, the impact of these three anxiety factors on the happiness of middle-aged individuals falls at an intermediate level. Notably, the direct influence of Buddhist culture on the happiness of middle-aged and elderly individuals surpasses its impact on young individuals. Particularly for the younger demographic, Buddhist culture emerges as more efficacious in alleviating the adverse effects of housing price pressure and unemployment anxiety on happiness. This observation could be elucidated by the heightened sense of ambition and determination for life improvement among the youth, leading them to place greater emphasis on personal efforts to enhance their well-being. Conversely, middle-aged and elderly individuals might perceive fewer opportunities for personal advancement, thus finding solace in the tranquility and comfort offered by Buddhist culture.

### 5.3 Number of friends

Having good friends can be a valuable source of support and aid during challenging periods, while also providing a platform to share joy. Individuals with a larger social circle may discover it’s more straightforward to manage stress, surmount obstacles, and achieve elevated levels of happiness. However, it’s important to note that social comparison within friendships can potentially amplify feelings of anxiety.

In this study, we categorized the sample into two distinct groups based on respondents’ number of friends—those falling below the 1/3 quantile and those surpassing the 2/3 quantile—and subsequently conducted regression analyses. The outcomes presented in Table 5, columns (6) and (7), unveil that for individuals boasting a larger circle of friends, the detrimental influence of housing price pressure, unemployment anxiety, and perceived inequality on their happiness is notably more pronounced. This trend could be attributed to the heightened social comparison that stems from having an increased number of friends. Conversely, among individuals with a substantial number of friends, the impact of Buddhist culture on their happiness appears to be relatively less potent. This observation can be rationalized by the notion that a strong support system from numerous friends can alleviate distress, thereby diminishing the necessity for heavy reliance on Buddhist culture. Consequently, the impact of one’s social circle on happiness demonstrates a “double-edged sword” effect.

### 5.4 Social insurance level

The level of social security is one of the factors that influence religious activities, as religious culture may offer certain forms of insurance (Dehejia et al., 2007). Medical insurance holds particular importance in safeguarding residents’ health and enhancing their risk resilience. In this study, the sample was divided into two groups based on whether respondents enjoyed medical insurance. The results in Table 5, columns (8) and (9), indicate that individuals without medical insurance not only face greater pressure from housing prices but also experience a more pronounced effect of Buddhist culture on their happiness. This phenomenon may be attributed to the fact that individuals with medical insurance possess stronger coping abilities regarding illness and medical expenses, have fewer concerns about health-related obstacles to housing purchase or relocation, and exhibit less uncertainty about the future. Conversely, residents without medical insurance face greater instability, making them more likely to seek spiritual or material support from Buddhist culture.

### 5.5 Health

Good health serves as the foundation for continuous striving and advancement. Individuals with good health tend to exhibit higher work efficiency, a more optimistic outlook, and stronger capabilities to withstand stress. Based on the respondents’ health conditions, this study divides the sample into two groups. A comparison of the results in Table 5, columns (10) and (11), reveals that individuals with good health contend with relatively milder adverse impacts resulting from housing price pressures and sentiments of inequality. Additionally, the influence of Buddhist culture on their happiness is comparatively less pronounced. In contrast, Buddhist culture demonstrates a stronger positive impact on the happiness of individuals with suboptimal health, possibly because those with poorer health are more prone to harboring pessimistic expectations and face limitations in life choices due to their health conditions. In this regard, Buddhist culture offers solace to their inner selves.

The heterogeneity analysis conducted above indicates that Buddhist culture exhibits a stronger safeguarding effect on happiness in rural areas, among middle-aged and elderly individuals, among those with fewer friends, individuals with lower levels of social security, and individuals with suboptimal health. This suggests that Buddhist culture has the capacity to partially compensate for the less favorable circumstances experienced by vulnerable groups in their daily lives, providing spiritual and emotional consolation.

## 6 Robustness checks

### 6.1 Replacement of main variables

Firstly, considering the multiple ways to measure subjective well-being, we replaced the dependent variable with “Overall, are you satisfied with your life situation?” Similarly, we transformed it into a binary variable: 1 for “satisfied” or “very satisfied” responses and 0 otherwise. The baseline regression results using this new dependent variable are presented in Table 6, column (1). Secondly, we used the number of Buddhist temples within a 200-kilometer radius of the respondents’ location as a measure of the degree of Buddhist culture density. The regression results for this variable are shown in Table 6, column (2). Thirdly, we employed the previous year’s *per capita* land transfer area as an instrumental variable for housing price pressure. Together with the instrumental variable for Buddhist culture, we conducted IV-probit regression, and the results are displayed in Table 6, column (3). Notably, the sign and significance of the main variables remained unchanged in these alternate specifications.

**Table 6:** Robustness checks.

	(1)	(2)	(3)	(4)	(5)	(6)	(7)	(8)
	Replace dependent variable	Replace independent variable	Use IV for housing prices	Ordered Logit	Cluster on city	Cluster on county	Irreligious subsample	First-difference estimator
Housing price pressure	−0.098***	−0.515***	−0.098***	−0.822***	−0.199***	−0.199***	−0.215***	−0.331**	(0.002)	(0.000)	(0.000)	(0.000)	(0.008)	(0.005)	(0.000)	(0.010)
Unemployment anxiety	−0.061***	−0.111***	−0.176***	−0.369***	−0.080***	−0.080***	−0.087***	−0.034***	(0.000)	(0.000)	(0.000)	(0.000)	(0.000)	(0.000)	(0.000)	(0.006)
Perceived inequality	−0.146***	−0.181***	−0.497***	−0.967***	−0.183***	−0.183***	−0.186***	−0.184***	(0.000)	(0.000)	(0.000)	(0.000)	(0.000)	(0.000)	(0.000)	(0.000)
Buddhist culture	0.014***	0.019***	0.016***	0.052***	0.013	0.013	0.013***	−0.009	(0.000)	(0.000)	(0.002)	(0.000)	(0.243)	(0.196)	(0.001)	(0.530)
HP pressure × Buddhist culture	0.083***	0.204***	0.504***	0.507***	0.135*	0.135**	0.158***	0.187**
	(0.001)	(0.000)	(0.000)	(0.000)	(0.058)	(0.042)	(0.000)	(0.017)
Unemployment × Buddhist culture	0.015**	0.022**	0.047***	0.085***	0.021**	0.021**	0.023***	0.016**
	(0.046)	(0.020)	(0.000)	(0.008)	(0.019)	(0.014)	(0.004)	(0.046)
Inequality × Buddhist culture	−0.009	−0.001	−0.010	−0.004	−0.001	−0.001	0.001	−0.004
	(0.116)	(0.881)	(0.322)	(0.874)	(0.929)	(0.922)	(0.843)	(0.693)
Other controls	Yes	Yes	Yes	Yes	Yes	Yes	Yes	Yes
Year FE	Yes	Yes	Yes	Yes	Yes	Yes	Yes	Yes
Observations	19,072	19,072	18,186	19,072	19,072	19,072	17,255	4,201

Coefficients in the table, except for those in column (4) which are directly obtained through maximum likelihood, represent average marginal effects. Values in parentheses are *p*-values; *p-value < 0.1, **p-value < 0.05, ***p-value < 0.01.

### 6.2 Alternative estimation methods

Firstly, we directly used the 1–5 scale to reflect subjective happiness, with each score corresponding to “very unhappy,” “unhappy,” “neutral,” “happy,” and “very happy,” respectively. We then conducted an ordered Logit regression as model (1), and the results are presented in Table 6, column (4). Secondly, to account for potential correlated disturbances among respondents located in close proximity, we clustered the standard errors at the city and county levels. The regression results using this clustered approach are reported in Table 6, columns (5) and (6). Importantly, the coefficients and significance of the main variables remained consistent across these alternative estimation methods.

### 6.3 Replacement of estimation sample

Firstly, to examine whether Buddhist culture also affects the happiness of non-religious individuals, we limited the sample to respondents who reported having no religious beliefs in the questionnaire. The results are presented in Table 6, column (7), showing that the coefficients and significance of the main variables remained unchanged. This suggests that the influence of Chinese Buddhist culture extends beyond Buddhists and has spillover effects on a wide range of non-religious residents (Clark and Lelkes, 2009). Secondly, to further reduce the impact of unobservable individual characteristics on happiness, we restricted the sample to respondents who were followed up in consecutive years and conducted a first-difference estimation. The results are shown in Table 6, column (8), and the coefficients and significance of the main variables remained consistent.

### 6.4 Addition of control variables

Firstly, we controlled for the Gini coefficient, registered unemployment rate, housing price-to-*per capita* disposable income ratio, and their interactions with the degree of Buddhist culture density to account for the objective impact of inequality, unemployment rate, and housing prices. The regression results are presented in Table 7, column (1).

**Table 7:** Robustness checks: adding controls.

	(1)	(2)
Housing price pressure	−0.160***	−0.076*	(0.000)	(0.060)
Unemployment anxiety	−0.076***	−0.053***	(0.000)	(0.000)
Perceived inequality	−0.184***	−0.119***	(0.000)	(0.000)
Buddhist culture	0.036**	0.043**	(0.038)	(0.025)
Housing price pressure × Buddhist culture	0.124***	0.070**
	(0.000)	(0.016)
Unemployment anxiety × Buddhist culture	0.017**	0.015*
	(0.032)	(0.060)
Perceived inequality × Buddhist culture	−0.000	−0.002
	(0.942)	(0.793)
Housing price	−0.148***	0.020	(0.006)	(0.742)
Housing price × Buddhist culture	−0.051	−0.125	(0.510)	(0.153)
Unemployment rate	−0.100***	−0.100***	(0.001)	(0.001)
Unemployment rate × Buddhist culture	0.061***	0.055***
	(0.000)	(0.000)
Gini index	−0.140***	0.035	(0.000)	(0.420)
Gini index × Buddhist culture	−0.007	−0.012	(0.546)	(0.288)
Other new controls	No	Yes
Year FE	Yes	Yes
Observations	18,034	17,340

Coefficients represent average marginal effects. Values in parentheses are *p*-values; *p-value < 0.1, **p-value < 0.05, ***p-value < 0.01.

Furthermore, we continued to include additional control variables, such as employment in state-owned enterprises, internet usage, height, weight, beauty, religious beliefs, afforestation of city, weather during the interview, job satisfaction, expectations of future social status, social trust, medical insurance coverage, number of friends, and province fixed effects. The regression results are reported in Table 7, column (2). The results in Table 7 demonstrate that the direction and significance of the main variable coefficients remained unchanged. Additionally, objective unemployment rate, income disparity, and housing prices also exerted a certain negative impact on subjective well-being. However, the protective effect of Buddhist culture on happiness was primarily manifested in alleviating residents’ subjective anxieties related to unemployment, inequality, and rising housing prices. In other words, the safeguarding effect of Buddhist culture on happiness was mainly achieved by regulating residents’ psychological and mental states.

### 6.5 Control the impact of other traditional Chinese culture

Concepts such as “relaxation” and “tranquility” can be found in other traditional Chinese cultures. Considering that Buddhism, Taoism, and Confucianism stand as three prominent examples of Chinese traditional culture, we took steps to control for the potential influence of Taoism and Confucianism, ensuring the reliability of our findings. We assessed the impact of Taoist culture by quantifying the number of Taoist temples per square kilometer in the city. The impact of Confucian culture is measured by genealogy density, as introduced by Chen et al. (2022), which is the number of genealogy books per 10,000 members of the population within the prefecture. Results presented in Table 8 demonstrate the robustness of our primary findings.

**Table 8:** Robustness checks: control the potential effects of other traditional Chinese culture.

	(1)	(2)
Confucian culture		−0.002		(−1.229)
Taoist culture	−0.054	−0.001	(−0.649)	(−0.009)
Housing price pressure	−0.942***	−0.988***	(−6.175)	(−6.239)
Unemployment anxiety	−0.376***	−0.381***	(−7.595)	(−7.587)
Perceived inequality	−0.862***	−0.852***	(−21.319)	(−20.726)
Buddhist culture	0.136	0.072	(1.171)	(0.595)
HP pressure × Buddhist culture	0.640***	0.635***	(5.165)	(5.060)
Unemployment × Buddhist culture	0.096***	0.101***	(2.664)	(2.773)
Inequality × Buddhist culture	−0.003	−0.003	(−0.094)	(−0.093)
Other controls	Yes	Yes
Year FE	Yes	Yes
Observations	19,072	18,422

Coefficients represent average marginal effects. Values in parentheses are *p*-values; *p-value < 0.1, **p-value < 0.05, ***p-value < 0.01.

### 6.6 Mitigate the problem of time lag in cultural leakage

We employ the number of Buddhist temples in 2014 to measure the density of Buddhist culture, but cultural leakage might have a lag. To alleviate this problem, we measure the density of Buddhist culture by the logarithm of temples classified as “National Key Buddhist Temples” within the area where a resident lives. The list of National Key Buddhist Temples was originally released on April 9, 1983, by the State Council of China. More information about these temples can be found on the website of the Buddhist Association of China.^11^ These key Buddhist temples hold longer histories and greater influence, which can reflect the heritage of Buddhist culture within the corresponding area and alleviate concerns about potential time lags in cultural leakage. Results in Table 9 confirm the robustness of our main findings.

**Table 9:** Robustness checks: use number of National Key Buddhist Temples as measure of Buddhist culture.

	(1)
	Happiness
Housing price pressure	−0.240^***^	(−6.087)
Unemployment anxiety	−0.079^***^	(−6.734)
Perceived inequality	−0.201^***^	(−21.662)
Ln(number of National Key Buddhist Temples)	0.023^***^	(3.433)
HP pressure × Ln(number of National Key Buddhist Temples)	0.285^***^	(5.074)
Unemployment × Ln(number of National Key Buddhist Temples)	0.041^***^	(3.447)
Inequality × Ln(number of National Key Buddhist Temples)	0.026^*^	(1.781)
Other controls	Yes
Year FE	Yes
Observations	19,072

Coefficients represent average marginal effects. Values in parentheses are *p*-values; *p-value < 0.1, **p-value < 0.05; ***p-value < 0.01.

### 6.7 Control the dimension of Buddhist faith

To ensure the robustness of our results, we use respondents’ answers to the question “whether you believe in Buddhism” as a proxy for Buddhist faith. We add Buddhist faith as controls and re-conduct regressions. The results presented in Table 10 demonstrate the consistency of our primary findings.

**Table 10:** Robustness checks: control the dimension of Buddhist faith.

	(1)		(2)
	Happiness		Happiness
Housing price pressure	−0.127^***^	Housing price pressure	−0.199^***^	(−4.302)		(−6.176)
Unemployment anxiety	−0.074^***^	Unemployment anxiety	−0.080^***^	(−7.321)		(−7.640)
Perceived inequality	−0.186^***^	Perceived inequality	−0.183^***^	(−23.621)		(−22.338)
Buddhist faith	0.016*	Buddhist culture	0.013^***^	(1.812)		(3.686)
HP pressure × Buddhist faith	0.082**	HP pressure × Buddhist culture	0.135^***^	(2.349)		(5.150)
Unemployment × Buddhist faith	0.033*	Unemployment × Buddhist culture	0.021^***^	(1.769)		(2.672)
Inequality × Buddhist faith	0.056	Inequality × Buddhist culture	−0.001	(1.272)		(−0.089)		Buddhist faith	−0.004			(−0.381)
Other controls	Yes		Yes
Year FE	Yes		Yes
Observations	19,072		19,072

Coefficients represent average marginal effects. Values in parentheses are *p*-values; *p-value < 0.1, **p-value < 0.05, ***p-value < 0.01.

### 6.8 Common method bias test

The issue of common method bias might be minimal concern for several reasons. First, our primary variables of interest in this study are drawn from disparate sources. Our measure of happiness is based on self-reported well-being, while the measurement of Buddhist culture pertains to the area of residence of the respondents. Moreover, we have taken measures such as employing instrumental variable regression to address endogeneity concerns, ensuring the consistency of our estimations. Second, many of our control variables are objective in nature, encompassing characteristics such as age, gender, and education. Consequently, the potential for shared variance between these objective indicators and subjective well-being is deemed relatively low. Third, any common variance among the control variables does not compromise the consistency of the estimation, especially considering our ample sample size for regression analysis.

Finally, we conducted Harman’s single factor test to fortify the reliability of our findings. This analysis yielded 13 factors, with a characteristic root exceeding one, and the first factor accounted for only 12% of the variance. This suggests that common method bias is not a significant issue.

## 7 Conclusions and discussions

The study delves into three primary factors that influence the well-being of Chinese residents: housing, employment, and fairness. In the midst of China’s economic transition, notably since the onset of the COVID-19 pandemic, residents’ apprehensions appear to have heightened, coinciding with an uptick in temple visits. Drawing upon data from the China Labor-force Dynamics Survey, this investigation reveals that the pressure of housing prices, anxieties about unemployment, and perceptions of inequality detrimentally affect residents’ subjective well-being. However, Buddhist culture emerges as a mitigating force, capable of ameliorating the adverse impacts of housing price pressure and unemployment anxiety, thereby offering a measure of happiness assurance. This protective influence is linked to the foundational tenets of Buddhist culture that empower individuals to approach challenges with equanimity and to navigate life’s uncertainties with serenity.

Buddhist culture serves to temper the erosion of job satisfaction, physical well-being, societal trust, and expectations for future social standing that stem from mounting housing and employment pressures. Additionally, it curtails the erosion of happiness arising from comparisons with others. Notably, the happiness assurance effect of Buddhist culture is particularly robust in rural areas, among middle-aged and elderly individuals, those with smaller social circles, those with more limited social security, and those contending with poorer health. This suggests that Buddhist culture partially compensates for the less favorable circumstances experienced by vulnerable groups in their daily lives, providing spiritual and emotional consolation.

The empirical findings carry substantial implications. For the average individual, religion can act as a source of spiritual solace amid present challenges, contributing to heightened personal happiness and improved resilience in facing real-world adversities. Following the COVID-19 pandemic, the anxiety levels among Chinese residents have notably increased. As a crucial element of traditional Chinese culture, the happiness insurance effect of Buddhist culture becomes more prominent. Psychotherapists may draw on certain aspects of religious philosophy in addressing mental disorders. Nonetheless, the path to a joyful existence is forged through determination. Confronting difficulties demands perseverance rather than shortcuts; in this context, Buddhist culture serves as an analgesic rather than a remedy. Ensuring physical well-being and nurturing positive social connections remain pivotal. Displaying resolve, sustaining progress, and embracing a trajectory of continuous growth represent the ultimate quest for happiness.

From a governmental perspective, there is potential to effectively steer religious culture towards fostering social harmony and promoting economic development (Oswald et al., 2015; DiMaria et al., 2020). Particular focus should be directed towards addressing the needs of vulnerable groups. In regions grappling with inadequate public service resources, there should be a concerted effort to enhance infrastructure development. It is crucial to recognize that elderly individuals often lack a strong voice in contemporary society. Therefore, establishing accessible platforms and channels for them to express themselves should be prioritized, while ensuring their well-being is given the due attention it deserves.

Chinese authorities maintain a dialectical perspective towards Buddhist culture. They discourage outdated ideological concepts and superstitious elements in Buddhist culture, while encouraging the utilization of its positive aspects to foster residents’ mental well-being, promote social harmony, cultural development, and economic growth. In October 2023, Xi Jinping^12^ thought on culture was introduced at a major national conference, which made requirements in “promoting the creative transformation and development of fine traditional Chinese culture.” From this point of view, religion culture can serve as a channel for international communication.

This paper also has several limitations. Using the number of joss sticks and candles burned at a temple as a measure of belief in Buddhist culture density is indeed a more ideal approach since devout disciples typically purchase these items, blessed by the temple, to express their faith. However, the unavailability of such data due to the lack of record-keeping and release by most temples poses a challenge, leading us to rely on suboptimal measures. Future research could increase the measurement dimension of “Buddhist faith” to obtain more interesting findings.

## Data availability statement

Publicly available datasets were analyzed in this study. This data can be found here: https://isg.sysu.edu.cn/node/353.

## Ethics statement

Ethical review and approval was not required for the study on human participants in accordance with the local legislation and institutional requirements. Written informed consent from the patients/participants or patients/participants' legal guardian/next of kin was not required to participate in this study in accordance with the national legislation and the institutional requirements.

## Author contributions

ST: Formal analysis, Investigation, Methodology, Writing – review & editing, Data curation, Funding acquisition, Resources, Supervision, Validation. PF: Methodology, Writing – original draft, Writing – review & editing, Conceptualization, Formal analysis, Investigation. SD: Methodology, Writing – original draft, Writing – review & editing. WS: Methodology, Formal analysis, Investigation, Writing – original draft, Preparation, Writing – review & editing.

## Footnotes

^1^ https://en.wikipedia.org/wiki/Great_Unity

^2^To describe people who reject the rat race and embrace a tranquil, apathetic life. https://en.wikipedia.org/wiki/Buddha-like_mindset. https://en.wikipedia.org/wiki/Tang_ping

^3^According to reports from the Worldwide Independent Network/Gallup International Association’s (WIN/GIA) global polls and the Pew Research Center in 2012, the percentage of citizens identifying as irreligious was low. In contrast, approximately 90% of the Chinese population identifies as atheist.

^4^Lots of American or European studies mentioned that Buddhism is the largest officially recognized religion in China (Wenzel-Teuber, 2017; Reem Pew Research Center, 2023; Religion in China, 2023; Albert and Maizland, n.d.; Pew Research Center, n.d.). This fact is also acknowledged by surveys in China (Li, 2017).

^5^We prioritize perceived inequality as one of our main variables of interest, as prior research has consistently demonstrated its stronger correlation with happiness, in contrast to objective inequality (Alesina et al., 2004; Schwarze and Härpfer, 2007; Schneider, 2016).

^6^ https://www.uri.org/kids/world-religions/buddhist-beliefs

^7^Many previous studies have utilized the China Labor-Force Dynamics Survey (CLDS) as their data source, as exemplified by Wang et al. (2017), Liu et al. (2019) and Ma et al. (2022).

^8^We also conducted an ordered-logit regression using the original five-point indicators as dependent variables. The estimation results are robust, which are available on request.

^9^The information concerning Buddhist temples is sourced from the National Religious Affairs Administration of China, which provides comprehensive data on nearly all temples in China, including details like name, location, and religious affiliation.

^10^Data on drought and flood disasters in different regions during the Ming and Qing dynasties is obtained from Weather China and the National Climate Center of China. These organizations have meticulously collected and organized historical materials, offering information on the severity of drought and flood in cities each year from 1470 to 1911.

^11^ https://www.chinabuddhism.com.cn/zdsy/

^12^Chairman of China.
